# Journal of Ovarian Research reviewer acknowledgement 2015

**DOI:** 10.1186/s13048-016-0215-1

**Published:** 2016-02-18

**Authors:** Sham S. Kakar, Stefano Palomba, Benjamin K. Tsang, David T. Curiel

**Affiliations:** Department of Physiology and Biophysics, University of Louisville, Louisville, KY 40202 USA; Department of Obstetrics, Gynecology and Pediatrics, Sterility Centre P. Bertocchi, Obstetrics and Gynecology Unit, A.O. S.Maria Nuova, IRCCS, Reggio Emilia and University of Modena, Reggio Emilia, Italy; Department of Obstetrics & Gynaecology, University of Ottawa and the Chronic Disease Program, Ottawa Hospital Research Institute, Ottawa, K1H 8 L6 Canada; Cancer Biology Division, School of Medicine, Washington University in St. Louis, St. Louis, MO 63108 USA

## Abstract

The editors of *Journal of Ovarian Research* would like to thank all of our reviewers who have contributed to the journal in Volume 8 (2015).

**Elham Abbasi**

Iran

**Nuzhat Ahmed**

Australia

**Juan Luis Alcazar**

Spain

**MŠ Alebić**

Croatia

**Giovanni Aletti**

Italy

**Simon Alfred**

Canada

**Adolfo Allegra**

Italy

**Carlo Alviggi**

Italy

**Mark Anderson**

United Kingdom

**Ritu Aneja**

United States of America

**Stefano Angioni**

Italy

**Fernando Anschau**

Brazil

**Amita Attlee**

United Arab Emirates

**Xiaochun Bai**

China

**Jay Baltz**

Canada

**Sharmila Bapat**

India

**Giovanni Battista La Sala**

Italy

**George R. Beck**

United States of America

**Susan Bellis**

United States of America

**Deepa Bhartiya**

India

**P Bhide**

United Kingdom

**Giuseppe Bifulco**

Italy

**Giorgio Bogani**

Italy

**Peter Bols**

Belgium

**Chinmoy Bose**

India

**Marcus Bosenberg**

Brazil

**Theodore Brown**

Canada

**Ariana Bruzzone**

Argentina

**David Buxton**

United States of America

**Yihua Cai**

United States of America

**Shelley Cargill**

United Kingdom

**Jvan Casarin**

Italy

**Livio Casarini**

Italy

**Sophie Catteau-Jonard**

France

**Juliana Celestino**

Brazil

**Rachel Chan**

Hong Kong

**Rachel Chan**

United Kingdom

**Shuo Chen**

China

**Zi-Jiang Chen**

China

**Ri-Cheng Chian**

Canada

**Indrajit Chowdhury**

United States of America

**Greg Christman**

United States of America

**Anita Chudecka-Glaz**

Poland

**Hugh Clarke**

Canada

**Fabrice Cognasse**

France

**Barbara Cometti**

Switzerland

**Pamela Constantinou**

United States of America

**Roberta Corona**

Belgium

**Giovanni Coticchio**

Italy

**Andrea Cupp**

United States of America

**Marek Cybulski**

Poland

**Chendil Damodaran**

United States of America

**Gennaro Daniele**

Italy

**Trinath Das**

United States of America

**John S. Davis**

United States of America

**Gaetano De Feo**

Italy

**Danny N. Dhanasekaran**

United States of America

**Martin Wanda A Dorsett**

American Samoa

**Joelle Dupont**

France

**Husamettin Ekici**

Turkey

**Dorraya El-Ashry**

United States of America

**Evelin Elia**

Argentina

**Sebastien Elis**

France

**Akmal El-Mazny**

Egypt

**Youssef Abo Elwan**

Egypt

**Kevin Eng**

United States of America

**Svend Engelholm**

Denmark

**Tze Kiong Er**

Taiwan

**Mike Fainzilber**

Iran

**Angela Falbo**

Italy

**Francesco Fanfani**

Italy

**Warren Foster**

Canada

**Wouter Froyman**

Belgium

**Fiona Furlong**

United Kingdom

**Dirk Geerts**

Netherlands

**Ancuta Gheorghisan-Galateanu**

Romania

**Fabio Ghezzi**

Italy

**Salvatore Gizzo**

Italy

**Norbert Gleicher**

United States of America

**Stefano Greggi**

Italy

**Umran Gulec**

Turkey

**Toshiyuki Hata**

Japan

**Yuka Hattori**

Japan

**Ioannis Hatzipetros**

Hungary

**Osamu Hiraike**

Japan

**Rashidi Batool Hossein**

Iran

**Ariel Hourvitz**

Israel

**Bunpei Ishizuka**

Japan

**Ciro Isidoro**

Italy

**Akira Iwase**

Japan

**Pooja Jagmohan**

Singapore

**Anders Jakobsen**

Denmark

**Lina Jia**

China

**Sangita Jindal**

United States of America

**Erica Johnson**

United States of America

**Masanori Kanemura**

Japan

**Sujata Kar**

India

**Adam Karpf**

United States of America

**Selvendiran Karuppaiyah**

United States of America

**John Kastelic**

Canada

**Shlomit Kenigsberg**

Canada

**A Khrunin**

Russian Federation

**Ki Hyung Kim**

South Korea

**Won Ho Kim**

South Korea

**Tadashi Kimura**

Japan

**Folgueira Maria Aparecida A Koike**

Brazil

**B Kotowicz**

United States of America

**Diane Krause**

United States of America

**Szyllo Krzysztof**

Poland

**Magdalena Kucia**

United States of America

**Miki Kushima**

Japan

**Antonio La Marca**

Italy

**Kevin Lam**

Hong Kong

**T N Vuong Lan **

Vietnam

**Florian Lang**

Germany

**Maria Laszczynska**

Poland

**Tien Le**

Canada

**Issam Lebbi**

Tunisia

**Keren Levanon**

Israel

**Marie-Claude Leveille**

Canada

**Julang Li**

Canada

**Monica Lipay**

Brazil

**Feng-Yuan Liu**

Taiwan

**Peishu Liu**

China

**Joshua Logan**

United States of America

**Emil Lou**

United States of America

**Zhen Lu**

United States of America

**Emilio Lucia**

Italy

**Jinfeng Ma**

China

**Mohammed Malik**

United States of America

**Vincenzo Dario Mandato**

Italy

**Marc Mansour**

Canada

**Claudia Marchetti**

Italy

**Artur Mayerhofer**

Germany

**Rajiv McCoy**

United States of America

**Ryan Meng**

China

**Janusz Menkiszak**

Poland

**Fakhroddin Mesbah**

Iran

**Anne-Marie Mes-Masson**

Canada

**Ioannis Messinis**

Greece

**Miller-Donald**

United States of America

**E Miralpeix**

Spain

**Gillian Mitchell**

Australia

**Ben Mol**

Australia

**Monica Montopoli**

Italy

**Barb Moreno**

Mexico

**Daria Morini**

Italy

**Giovanni Morrone**

Italy

**P. B. Mullan**

United Kingdom

**Carine Munaut**

Belgium

**Giovanna Muscogiuri**

Italy

**Ercan Cihangir Mutlu**

Turkey

**Mani Nassir**

Germany

**Kaei Nasu**

Japan

**Kazem Nejati**

Iran

**Ceana Nezhat**

United States of America

**Ernest Ng**

Hong Kong

**Alessia Nicoli**

Italy

**Warren Nothnick**

United States of America

**Kiyoshi Okada**

Japan

**Renato Oliveira**

Brazil

**Aki Oride**

Japan

**Francesco Orio**

Italy

**Makoto Orisaka**

Japan

**Raoul Orvieto**

Israel

**Yutaka Osuga**

Japan

**Johannes Ott**

Austria

**Emre Goksan Pabuccu**

Turkey

**George Pados**

Greece

**Stefano Palomba**

Italy

**Renato Pasquali**

Italy

**Manish Patankar**

United States of America

**Bibbin Paul**

United States of America

**Michael Pearl**

United States of America

**Emanuele Pelosi**

United States of America

**Chun Peng**

Canada

**Merviel Philippe**

France

**Moorthy Palanimuthu Ponnusamy**

United States of America

**Luis Quiñones**

Chile

**Christina Rasmussen**

Denmark

**Mariusz Ratajczak**

United States of America

**Suman Rice**

United Kingdom

**Carmen Romero**

Chile

**Carlo Maria Rotella**

Italy

**Debarshi Roy**

United States of America

**Rosaria Ruggeri**

Italy

**M Ram Sairam**

Canada

**Amany Salama**

Egypt

**Mauricio Salcedo**

Mexico

**BO Saleh**

Iraq

**Sanada Sakiko**

Japan

**Jindal Sangita**

United States of America

**Carol Sartorius**

United States of America

**Wlodzimierz Sawicki**

Poland

**Em Schmelz**

United States of America

**Matthew Schultz**

United States of America

**Kok-Min Seow**

Taiwan

**Masayuki Shimada**

Japan

**Tadahiro Shoji**

Japan

**Yimin Shu**

United States of America

**Santhi Silambanan**

India

**Ajay Singh**

United States of America

**Rajesh Singh**

United States of America

**Rajiv Singla**

India

**Marc-Andre Sirard**

Canada

**Michelle KY Siu**

Hong Kong

**Steven C Smith**

Swaziland

**Vinayak Smith**

Australia

**Johan Smitz**

Belgium

**Rakesh Srivastava**

United States of America

**Sherri Stewart**

United States of America

**Norihiro Sugino**

Japan

**Toru Sugiyama**

Japan

**Suman Suman**

United States of America

**Pierosandro Tagliaferri**

Italy

**Toshifumi Takahashi**

Japan

**Fuminori Taniguchi**

Japan

**Vasilios Tanosv**

Cyprus

**Hakan Timur**

Turkey

**Alison Ting**

United States of America

**Antoine Torre**

France

**Filippo Maria Ubaldi**

Italy

**Stefano Uccella**

Italy

**Guido Valente Valente**

Italy

**Bruno van Herendael**

Belgium

**Engelina van Houten**

Netherlands

**Barbara Vanderhyden**

Canada

**Lukas Vrba**

United States of America

**Qi Wang**

United States of America

**Robert Weinberg**

United States of America

**Stephanie B Wheeler**

Austria

**Marie Louise Wissing**

Denmark

**Jerzy Wydmanski**

Poland

**Yang Xiaokui**

China

**Kai Xue**

China

**Takashi Yazawa**

Japan

**Bulent O Yildiz**

Turkey

**Yoshihito Yokoyama**

Japan

**Er Leong Yong**

Singapore

**Jian Zhang**

China

**Yue Zhao**

China

**Chun Zhou**

United States of America

**R.S. Zimmerman**

United States of America

**Surekha Zingade**

India

**Fulvio Zullo**

Italy

**Enrico Zupi**

Italy

